# Traditional Chinese medicine therapies for patients with knee osteoarthritis: A protocol for systematic review and network meta-analysis

**DOI:** 10.1097/MD.0000000000029404

**Published:** 2022-07-15

**Authors:** Boyu Wu, Lei Yang, Liying Chen, Lu Ma, Yantao Guo

**Affiliations:** a The First Affifiliated Hospital of Hunan University of Chinese Medicine, Changsha, Hunan Province, China; b Hunan University of Chinese Medicine, Changsha, Hunan Province, China; c Zhejiang Chinese Medical University, Hangzhou, Zhejiang, China.

**Keywords:** knee osteoarthritis, network meta-analysis, protocol, traditional Chinese medicine

## Abstract

**Background:**

Knee osteoarthritis (KOA) is a common cause of chronic musculoskeletal pain and disability as well as a socioeconomic burden on healthcare services globally. Numerous clinical trials indicated that traditional Chinese medicine (TCM) may effectively improve the clinical symptoms of KOA patients. However, the comparative efficacy and safety of different TCM therapies in patients with KOA is not yet clear. In order to evaluate the efficacy and safety of TCM for KOA, we will conduct a systematic review and network meta-analysis on the existing randomized controlled trials (RCTs).

**Methods:**

A systematic literature search will be conducted in PubMed, Web of Science, Embase, EBSCO, Cochrane Library, China National Knowledge Infrastructure, Wanfang, Chinese Biomedical Literature Database, and the VIP Database for Chinese Technical Periodicals up to February 2022 to identify the relevant RCTs. The primary outcomes are visual analog scale, Western Ontario and McMaster Universities Osteoarthritis Index, Lysholm score, and Lequesne index. Secondary outcomes include the total clinical effective rate and adverse events. Study quality will be evaluated using the Cochrane risk of bias tool (RoB 2.0) for RCTs. Data analysis will be performed using Stata and WinBUGS. The quality of evidence will be assessed using the Grades of Recommendations Assessment Development and Evaluation.

**Results:**

The results of this study will be submitted to a peer-reviewed journal for publication.

**Conclusions:**

This study will provide evidence-based medical evidence for the treatment of KOA with TCM therapies and offer better assistance for clinical practice.

**Protocol registration number:**

INPLASY202230008.

## 1. Introduction

Knee osteoarthritis (KOA) is a common chronic degenerative disease characterized by progressive damage of articular cartilage, subchondral bone remodeling, and secondary synovial inflammation.^[1,2]^ It is one of the leading causes of disability, seriously affecting patients’ quality of life.^[3]^ KOA affects >250 million people worldwide,^[4]^ and will increase substantially with aging of the global population and the obesity epidemic.^[5]^ Therefore, KOA has emerged as a main global health problem and resulted in a substantial economic burden on the health care systems.^[6]^

According to international guidelines,^[7,8]^ the therapeutic drugs for KOA mainly include nonsteroidal anti-inflammatory drugs, analgesics, opioids, and steroid injections, which only alleviate symptoms but fail to block disease progression. Moreover, these drugs may lead to a series of side effects, including gastrointestinal bleeding, hepatic and renal toxicity, and cardiovascular complications, and hence long-term clinical use has been limited.^[9,10]^ Due to the limitations and adverse effects associated with the existing pharmacological treatments, research into therapies for KOA in complementary and alternative medicine (CAM) is receiving greater attention.^[11–13]^

Traditional Chinese medicine (TCM) is one of the most commonly used CAM modalities, including Chinese herbal medicine, acupuncture, moxibustion, tuina (Chinese massage), cupping, and other forms, which may be administered alone or in combination.^[14]^ Its efficacy and safety have been confirmed through long-term clinical practice. Presently, accumulating evidence indicates that TCM can effectively improve the clinical symptoms in KOA patients with a high degree of safety and few side effects.^[15–17]^ However, due to a relatively small amount of direct head-to-head comparison studies between different types of TCM therapies, the comparative outcomes between TCM therapies are still inconclusive.

Network meta-analysis (NMA) has been widely used in medical research, as it allows multiple interventions to be compared and ranked, overcoming the limitations of conventional pairwise meta-analyses.^[18]^ In this study, we will conduct an NMA to evaluate the efficacy and safety of various TCM therapies adopted in KOA treatment. We believe this work will provide evidence-based medical evidence for the treatment of KOA with TCM therapies and offer better assistance for clinical practice.

## 2. Methods

This systematic review and NMA protocol conform to the Preferred Reporting Items for Systematic Reviews and Meta-Analyses statement for Protocols guidelines.^[19]^ Our protocol has been registered on INPLASY with the registration number INPLASY202230008.

### 2.1. Eligible criteria

#### 2.1.1. Types of studies.

All randomized controlled trials on TCM therapies for KOA will be included. The languages of publication will be restricted to Chinese and English. We will exclude the following types of publications: case reports, case series, letters, comments, conference abstracts, reviews, animal studies, and studies with incomplete data.

#### 2.1.2. Types of participants.

We will include adult participants (≥18 years) diagnosed (based on radiographic evidence and clinical criteria) with KOA, irrespective of country, race, and gender.

#### 2.1.3. Type of interventions and comparisons.

In the experimental group, any form of TCM therapies will be included, such as Chinese herbal medicine, acupuncture, moxibustion, tuina (Chinese massage), cupping therapy, etc. In routine clinical practice, clinicians usually use a combined therapy to treat KOA, so the combination of different TCM therapies will also be included. Patients in the control group were treated with western medicine or placebo. Additionally, therapies combining western medicine with TCM will be excluded.

#### 2.1.4. Types of outcome measures.

The primary outcome measures were: visual analog scale, Western Ontario and McMaster Universities Osteoarthritis Index (WOMAC), Lysholm score, and Lequesne index. The secondary outcome measures were: total clinical effective rates and adverse events. Other outcomes will also be assessed if necessary.

### 2.2. Search strategy

Five English databases (PubMed, Web of Science, EMbase, EBSCO, and Cochrane Library) and 4 Chinese databases (China National Knowledge Infrastructure, Wanfang, Chinese Biomedical Literature Database, and the VIP Database) will be searched for randomized controlled trials published from the database inception through February 2022. We will use a combination of MeSH terms and free text words to identify relevant studies. The search strategy will be adjusted for the syntax appropriate for each database. The search strategy for PubMed is provided in Supplemental Material (Supplemental Digital Content, http://links.lww.com/MD/G884).

### 2.3. Study selection

**Figure 1.**
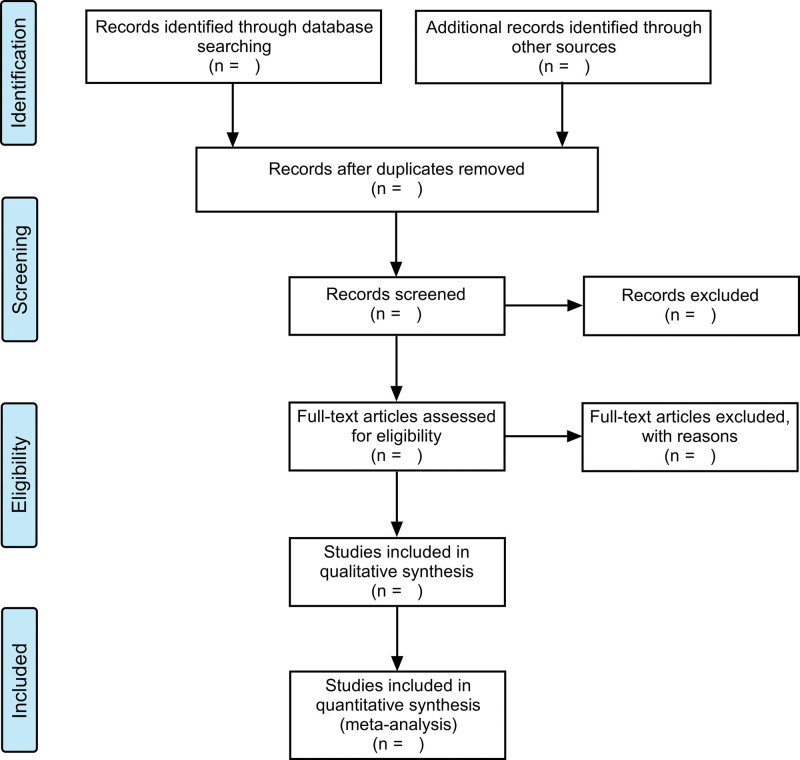
Flowchart of study selection.

All citations generated through the literature search will be imported into Endnote X9, and duplicates will be removed. Two reviewers will independently screen the titles and abstracts of all retrieved studies. After the initial screening, we will review full-text publications of potential studies. Any discrepancies between the 2 reviewers will be resolved by a third reviewer. The study selection process will be represented in Preferred Reporting Items for Systematic Reviews and Meta-Analyses flow diagram (Fig. 1).

### 2.4. Data extraction

Two reviewers will independently extract data from each study using a standard data abstraction sheet. Extracted items will include first author, publication year, region/country, sample size, mean age, sex distribution, study design, detailed interventions, follow-up time, and outcomes. Disagreements regarding data extraction will be resolved by discussion and consensus with a third reviewer.

### 2.5. Quality assessment

We will use the Cochrane risk of bias tool (RoB 2.0) for quality assessment.^[20]^ This tool consists of 5 domains (randomization process, intended interventions, missing outcome data, the measurement of the outcome, and the selection of reported results). Each domain is classified as low risk, some concerns, and high risk. Two reviewers will use RoB 2.0 to evaluate all included studies, and a third reviewer will serve as a consultant to resolve the disagreement if necessary.

### 2.6. Statistical analysis

#### 2.6.1. Pairwise meta-analysis.

We will perform the pairwise meta-analysis on direct comparisons with STATA 15.0. Odds ratio with 95% confidence intervals (CIs) will be applied for dichotomous outcomes, while mean difference or standard mean difference with 95% CI will be calculated for continuous variables. Heterogeneity among studies will be assessed using the I-square statistic.^[21]^ A random-effects model will be used if *I^2^* is >50%, otherwise a fixed-effects model will be applied.

#### 2.6.2. Network meta-analysis.

We will conduct the NMA on indirect comparisons with WinBUGS 1.4.3 and STATA 15.0. A random-effects model will be used to perform the NMA, considering the anticipated clinical heterogeneity among studies. The Brooks–Gelman–Rubin method will be used to examine the model convergence.^[22]^ Node-splitting method will be used to estimate the inconsistency by comparing the direct evidence with the indirect evidence.^[23]^ To obtain the ranking probability of the different treatments, the surface under the cumulative ranking curve (SUCRA) will be calculated.^[24]^

### 2.7. Subgroup and sensitivity analyses

If heterogeneity or inconsistency among the studies is detected, subgroup analysis will be performed according to the sample size, age group, patient severity, treatment duration, and other relevant parameters. We will also conduct sensitivity analyses by removing each study 1 at a time to evaluate the stability of the results.

### 2.8. Publication bias

In pairwise comparisons with at least 10 studies, funnel plots will be used to examine publication bias and the effects of small studies.^[25]^ We will apply Egger test to test for funnel plot asymmetry.^[26]^

### 2.9. Quality of evidence

We will use the Grades of Recommendations Assessment Development and Evaluation approach to rate the quality of evidence of the NMA results.^[27]^ The Grades of Recommendations Assessment Development and Evaluation approach grades the quality of evidence as very low, low, moderate, or high.

### 2.10. Ethics and dissemination

The study does not require ethical approval as it does not involve the collection of private information or affect the patients’ right.

## 3. Discussion

KOA is a common degenerative joint disease that not only causes pain and physical disability but also poses a substantial economic burden on society.^[1–4]^ TCM has been recognized as one of the typical representatives of CAM, which are attracting increasing international attention. Meanwhile, there is increasing evidence that multiple TCM therapies can be safe and effective in patients with KOA. Meanwhile, an increasing number of clinical and experimental studies have confirmed that TCM can effectively relieve the pain of KOA and enhancing patients’ quality of life. Currently, several conventional pairwise meta-analyses have investigated the comparative efficacy and safety of single TCM therapies for KOA.^[16,28,29]^ However, no NMA has been performed to evaluate the comparative efficacy and safety of all the available TCM therapies. Therefore, the aim of our NMA is to determine the safest and most effective TCM therapy for KOA, and to fill gaps in extant literature. We expect that the results of this NMA will facilitate the decision-making by clinicians in the treatment of patients with KOA with TCM therapies.

However, this study also has some limitations. First, all included literature in the NMA are Chinese and English language publications, which may cause a certain degree of selective reporting and publication bias. Furthermore, it is difficult to rule out heterogeneity completely because of the differences in specific prescription, treatment frequency, follow-up time, etc.

## Author contributions

Conceptualization: Boyu Wu, Yantao Guo.

Formal analysis: Boyu Wu, Lei Yang, Yantao Guo.

Methodology: Boyu Wu, Lei Yang, Lu Ma.

Project administration: Lei Yang, Lu Ma.

Software: Boyu Wu, Lu Ma, Liying Chen.

Writing – original draft: Boyu Wu, Lei Yang, Liying Chen.

Writing – review & editing: Boyu Wu, Yantao Guo.

## Supplementary Material


