# Supplementary material for: Traditional Chinese medicine therapies for patients with knee osteoarthritis: A protocol for systematic review and network meta-analysis
Source: Medicine (Baltimore). 2022 Jul 15;101(28):e29404. doi: 10.1097/MD.0000000000029404 (PMC11132338; doi:10.1097/MD.0000000000029404)
Supplement: Supplementary file 1 [file medi-101-e29404-s001.pdf]

| #ID | Topic or intervention | Query |
|---|---|---|
| #1 | Disease | ((("Osteoarthritis, Knee"[Mesh]) OR ((((Knee[Title/Abstract]) OR (Knees[Title/Abstract])) OR ("Knee Joint"[Mesh])) AND ("Osteoarthritis"[Mesh]))) OR ((((Knee[Title/Abstract]) OR (Knees[Title/Abstract])) OR ("Knee Joint"[Mesh])) AND ((((Osteoarthritis[Title/Abstract]) OR (Osteoarthritides[Title/Abstract])) OR (Arthritis[Title/Abstract])) OR (Arthritides[Title/Abstract])))) OR (((Knee OA[Title/Abstract]) OR (Knees OA[Title/Abstract])) OR (KOA[Title/Abstract])) |
| #2 | intervention | (((((("Medicine, Chinese Traditional"[Mesh]) OR "Drugs, Chinese Herbal"[Mesh]) OR ( "Acupuncture"[Mesh] OR "Acupuncture, Ear"[Mesh] OR "Acupuncture Therapy"[Mesh] )) OR "Electroacupuncture"[Mesh]) OR "Moxibustion"[Mesh]) OR "Cupping Therapy"[Mesh]) OR ((((((((((((((((Chinese Medicine[Title/Abstract]) OR (Chinese Herbal[Title/Abstract])) OR (Acupuncture[Title/Abstract])) OR (Fire needling[Title/Abstract])) OR (Warm needling[Title/Abstract])) OR (Tuina[Title/Abstract])) OR (Chinese massage[Title/Abstract])) OR (Cupping[Title/Abstract])) OR (Scrapping[Title/Abstract])) OR (Gua Sha[Title/Abstract])) OR (Bloodletting[Title/Abstract])) OR (Acupoint sticking[Title/Abstract])) OR (Acupoint injection[Title/Abstract])) OR (Herb fumigation[Title/Abstract])) OR (Herb soaking[Title/Abstract])) OR (Tai Chi[Title/Abstract])) OR (Qigong[Title/Abstract])) |
| #3 | Study design | ((((Randomized Controlled Trial[Publication Type]) OR (Randomized[Title/Abstract])) OR (Randomised[Title/Abstract])) OR (Randomization[Title/Abstract])) OR (Randomisation[Title/Abstract]) |
| #4 | Final query | #1 AND #2 AND #3 |
